# Supplementary material for: Computational simulation to assess patient safety of uncompensated COVID-19 two-patient ventilator sharing using the Pulse Physiology Engine
Source: PLoS One. 2020 Nov 25;15(11):e0242532. doi: 10.1371/journal.pone.0242532 (PMC7688119; doi:10.1371/journal.pone.0242532)
Supplement: S1 File — (PDF) [file pone.0242532.s001.pdf]

S1 File

Multi-patient ventilation design

The lumped-parameter models in the Pulse Physiology Engine are shown in S1 Fig for the respiratory system and the ventilator. Two patients respiratory circuits are connected to a single ventilator for transient circuit analysis. The air pressure, flow, and volume and substance amounts are calculated for each time step. This physics-based approach provides an flexible methodology for changing the resistors and/or capacitors in the lumped-parameter models to represent disease states, such as ARDS.

S1 Fig. The combined closed-loop lump-parameter fluid circuit for simulating multi-patient ventilation. A new entire system state is calculated every two ms. The Pulse dynamic circuit solver and transporter is leveraged to ensure sound physics-based results with conservation of energy and mass. Not explicitly shown are interactions with all other Pulse physiological systems, most notably, the alveolar-capillary partial pressure gradient diffusion gas exchange with the cardiovascular system.

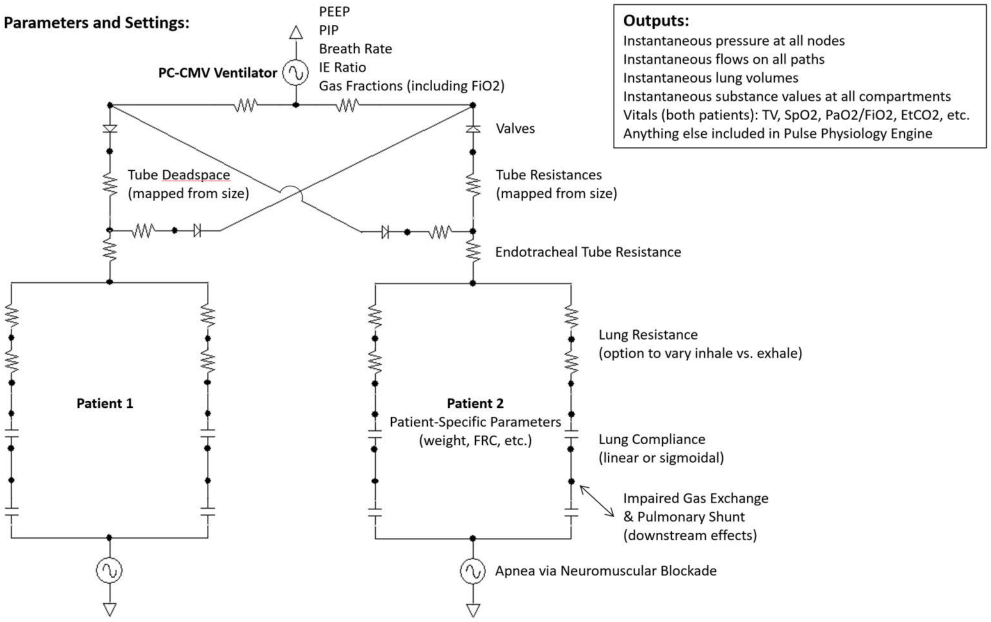

Diffusion impairment factor design

To implement the COVID-19 disease state, the Diffusion Impairment Factor (DIF) was calculated. The factor was mapped to the cardiovascular circuit in Pulse at the intersection of the cardiovascular and respiratory circuits, as shown in S2 Fig.

S2 Fig. The Diffusion Impairment Factor (DIF) implementation on the pulmonary section of the cardiovascular circuit in the Pulse Physiology Engine. The DIF severity is mapped to cardiovascular circuit modifiers to hinder alveolar gas exchange and increase pulmonary shunting.

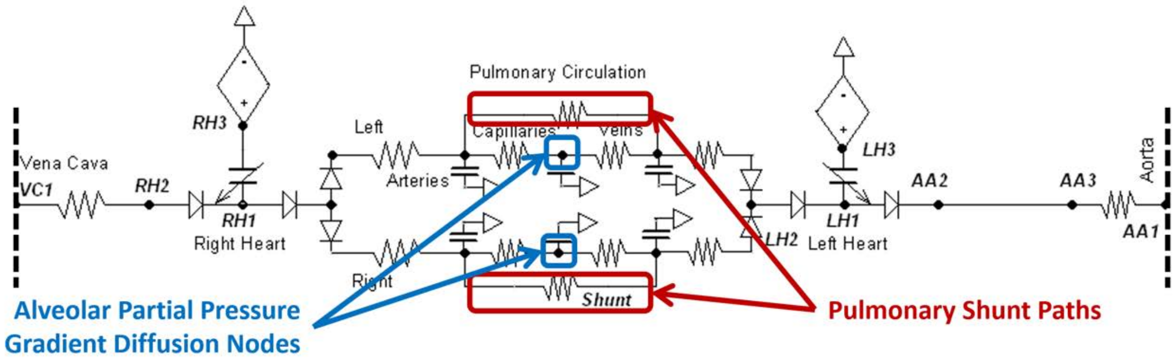

The pulmonary shunt resistance and alveolar surface area are both reduced by a factor (y) based on severity (x [0 to 1]) following the exponential decay equation (Eq (1)).

$$y = 10^{\ln\left(x\frac{b}{a}\right)+\ln(a)}, \qquad (1)$$

Where, a is 1.0 and b is 0.1 for the alveolar surface area and a is 1.0 and b is 0.01 for the pulmonary shunt resistance change.

Patient exclusion criteria

As noted in the Methods section, the patients were initially stabilized on a single ventilator. By varying the DIF and modifying the compliance, the patients were created with various disease states. Some of these patients were unable to meet our scoring criteria for multi-patient ventilation on an individual ventilator. Therefore, they were excluded from further analysis, so prevent noise in the data. S1 Table shows the percentage of patients able to meet the oxygen saturation scoring criteria of at least 89%.

S1 Table. Patient Exclusion Results. Patients with higher DIF severities require a higher ventilator FiO_2 setting to achieve positive (green) outcomes based on SpO_2. The total fraction of patients that are included for multi-patient simulation analysis are listed in the third column. The minimum FiO_2 setting is 0.21 (natural air).

**DIF**	**Mean Minimum FiO_2 for $SpO_2 > 89\%$**	**Fraction of Patients Achieving $SpO_2 > 89\%$**
0.3	0.21	100%
0.4	0.21	100%
0.5	0.23	100%
0.6	0.27	100%
0.7	0.46	100%
0.8	0.95	29%
0.9	N/A	0%

Simulation pseudocode

For this study, we chose the following ranges (Minimum, Maximum, Step):

- PEEP (cmH_2O) : Minimum:10, Maximum:20, Step Size:5
- Compliance (mL/cmH_2O) : Minimum:10, Maximum:50, Step Size:10
- DIF (%): Minimum:30, Maximum:90, Step Size:10

This generates a total of 12,642 unique simulations to execute. The execution loop is shown in Algorithm 1.

Algorithm 1: The Pulse multi-patient execution pseudocode for this investigation.

Set patient resistances to 5 cmH_2O-s/L;
Set ventilator RR to 20 bpm and I:E Ratio to 1:2;
for *Range of PEEP* **do**
    Set ventilator PEEP;
    for *Range of average compliances* **do**
        Calculate the optimal PIP for TV to 6 mL/kg based on current PEEP and average compliance;
        Calculate a range of inner level compliances for each patient;
        for *Calculated patient compliances* **do**
            Set patient compliances;
            for *All combinations of patient DIF* **do**
                Set Patient DIFs;
                while *any patient SpO_2 < 89% (pass) and ventilator FiO_2 < 100% (fail)* **do**
                    Increase ventilator FiO_2 and simulate until SpO_2 is stable for 10s;
                end
                Store results;
            end
        end
    end
end

Oxygen saturation stability is determined by storing the lowest SpO_2 of all engines as a baseline value at a specified time. For each subsequent time step, we sample the lowest SpO_2 calculated and determine the percent difference with the baseline value. The SpO_2 is considered stable when the percent different is less than 0.25% for 10s. Once a stable SpO_2 value is achieved, the FiO_2 setting on the ventilator is adjusted accordingly:

- If the SpO_2 is trending down and $< 85\%$, FiO_2 is increased by 10%
- If the SpO_2 is trending down and $\geq 85\%$, FiO_2 is increased by 2.5%
- If the SpO_2 is trending up and $< 88\%$, FiO_2 is increased by 5%
- If the SpO_2 is trending up and $> 88\%$, FiO_2 is increased by 1%

Pulse Physiology Engine architecture

Architecturally, a single instance of the multiplex ventilation engine accepts a list of simulations to run. Each simulation in the list contains the compliance, resistance, and impairment for each patient, and the ventilator settings for the simulation. As each engine simulates, it creates a log of the simulation and a CSV (Comma Separated Values) file containing the simulation results for each timestep. When both patients within the engine reach final homeostasis, the final values of interest will be appended to the simulation list for the entire experiment. When all engines have completed, the simulation list is output as a JSON (JavaScript Object Notation) file.

Pulse Physiology Engine data model

Pulse implements its data model (model definitions) via Google Protocol Buffers (GPB). This provides the ability to support instantiation and control of Pulse engines from

various programming languages and network protocols. GPB also provides the ability to read and write data in the popular JSON format for readability and import into popular data analysis libraries. The GBP definitions used by the Multiplex Ventilation Engine can be found here, and below is an example of the data properties computed for a single patient.

Patient State JSON:

{
"ID": 0,
"Compliance_mL_Per_cmH$_2$O": 10,
"Resistance_cmH$_2$O_s_Per_L": 5,
"ImpairmentFraction": 0.40000000596046448,
"RespirationRate_Per_min": 20,
"IERatio": 0.5,
"PEEP_cmH$_2$O": 10,
"PIP_cmH$_2$O": 55,
"FiO$_2$": 0.21,
"AirwayFlow_L_Per_min": -384.06825814934518,
"AirwayPressure_cmH$_2$O": 1043.4194836658687,
"AlveolarArterialGradient_mmHg": 45.619370140146856,
"ArterialCarbonDioxidePartialPressure_mmHg": 39.819789561334652,
"ArterialOxygenPartialPressure_mmHg": 75.85119486614299,
"CarricoIndex_mmHg": 367.5726868121024,
"EndTidalCarbonDioxidePressure_mmHg": 20.822935895891064,
"IdealBodyWeight_kg": 75.3,
"MeanAirwayPressure_cmH$_2$O": 25,
"OxygenationIndex": 11.003491209618154,
"OxygenSaturation": 0.9603982517625641,
"OxygenSaturationIndex_mmHg": 6.3974838958631794,
"SFRatio": 4.6503130439307032,
"ShuntFraction": 0.087940097305858056,
"TidalVolume_mL": 449.99997579831688,
"TotalLungVolume_mL": 2680.9772230837052,
"AchievedStabilization": true,
"OxygenSaturationStabilizationTrend": -0.11851467873102428
}

Resource requirements

Pulse has a small memory footprint ($<$ 10MB of RAM) and simulates at 10x real-time while using 100% of one average CPU core. Since the multiplex ventilation engine contains two patients, but with a shared respiratory circuit it runs around 6x real-time using 100% of one CPU core. Pulse is not a good candidate for multi-threaded execution of the physiology analysis as the individual system models must be executed in series. A runner was created to run each simulation in the simulation list on its own CPU core via threads. For our study, we employed an Alienware Area-51 R6 with dual processors (16-Cores each, overclocked up to 3.6GHz) and 64GB of RAM.

Graphical user interface

As part of this effort, we created bindings to instantiate and control the multiplex engine using the Python programming language. We were able to create and deploy a Jupyter Notebook that provides a simple interface for end users to customize their own patients (via compliance, resistance and impairment factors) and control the ventilation

while viewing simulation results in real-time. The Jupyter Notebook GUI can be found in our gitlab repository (`https://gitlab.kitware.com/physiology/jupyter`) and is shown in S3 Fig.

S3 Fig. Jupyter Notebook User Interface. The user interface allows for patient customization of the disease state and the ventilator settings for multi-patient ventilation. Results of the simulation are shown in the lower portion of the simulation.

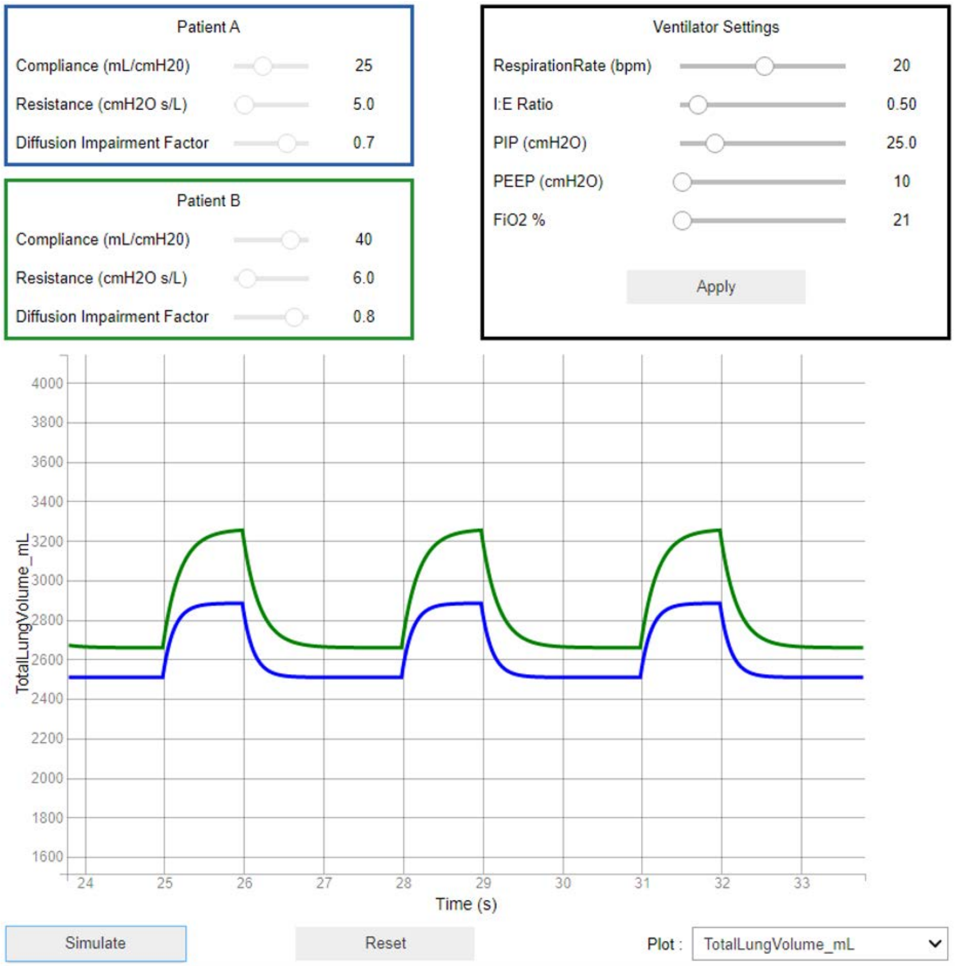

Source code and associated data

The Pulse Physiology Engine, the multiplex ventilation engine for Pulse, and the GUI are open source under the Apache 2.0 software license. All code can be obtained from our gitlab repository (`https://gitlab.kitware.com/physiology/engine/-/tags/STUDY_MULTIPLEX_VENTILATION_1_0_0`), and can be used to generate all of the final study data. To build the engine, follow the build instructions provided on the repository ReadMe: `https://gitlab.kitware.com/physiology/engine/-/blob/3.x/ReadMe.md` All data used in this study, as well as run instructions for the multiplex ventilation engine can be found in the multiplex ventilation engine ReadMe: `https://gitlab.kitware.com/physiology/engine/-/blob/3.x/src/cpp/study/multiplex_ventilation/ReadMe.md`.
